# Mesenchymal Stem Cells from Patients with Rheumatoid Arthritis Display Impaired Function in Inhibiting Th17 Cells

**DOI:** 10.1155/2015/284215

**Published:** 2015-03-31

**Authors:** Yue Sun, Wei Deng, Linyu Geng, Lu Zhang, Rui Liu, Weiwei Chen, Genhong Yao, Huayong Zhang, Xuebing Feng, Xiang Gao, Lingyun Sun

**Affiliations:** ^1^Department of Rheumatology and Immunology, The Affiliated Drum Tower Hospital of Nanjing University Medical School, Nanjing, Jiangsu 210008, China; ^2^Key Laboratory of Model Animal for Disease Study, Model Animal Research Center, Nanjing University, Nanjing, Jiangsu 210000, China

## Abstract

Mesenchymal stem cells (MSCs) possess multipotent and immunomodulatory properties and are suggested to be involved in the pathogenesis of immune-related diseases. This study explored the function of bone marrow MSCs from rheumatoid arthritis (RA) patients, focusing on immunomodulatory effects. RA MSCs showed decreased proliferative activity and aberrant migration capacity. No significant differences were observed in cytokine profiles between RA and control MSCs. The effects of RA MSCs on proliferation of peripheral blood mononuclear cells (PBMCs) and distribution of specific CD4^+^ T cell subtypes (Th17, Treg, and Tfh cells) were investigated. RA MSCs appeared to be indistinguishable from controls in suppressing PBMC proliferation, decreasing the proportion of Tfh cells, and inducing the polarization of Treg cells. However, the capacity to inhibit Th17 cell polarization was impaired in RA MSCs, which was related to the low expression of CCL2 in RA MSCs after coculture with CD4^+^ T cells. These findings indicated that RA MSCs display defects in several important biological activities, especially the capacity to inhibit Th17 cell polarization. These functionally impaired MSCs may contribute to the development of RA disease.

## 1. Introduction

The bone marrow microenvironment contains a population of self-renewing stromal stem cells, referred to as mesenchymal stem cells (MSCs), which provide support for haematopoietic progenitor cells [1]. MSCs are better known for their multilineage differentiation and immunomodulatory effects [2]. These cells possess significant chemotactic ability to migrate to sites of injury and inflammation, where they could exert anti-inflammatory and antiproliferative effects [3]. Thus, MSCs hold great promise for treating various diseases including autoimmune diseases (AD).

Rheumatoid arthritis (RA) is a prototypical AD and affects about 1% of the population worldwide [4]. The pathological processes of RA are largely played out by cells from the adaptive immune response including B and T cells, among which CD4^+^ T helper cells are one of the key actors. Many studies demonstrated that the imbalance between Th17 and regulatory T cells (Treg) plays an important role in the development and progression of RA [5, 6]. Our previous studies have demonstrated that the frequencies of circulating T follicular helper (Tfh) cells were markedly increased in RA patients and positively correlated with the level of autoantibodies, implying that Tfh cells may also participate in RA pathogenesis [7].

As suggested by our study and others [8–10], allogeneic MSC transplantation may provide some benefits to refractory RA patients. MSCs are able to inhibit the proliferation of activated peripheral blood mononuclear cells (PBMCs) and T lymphocytes [11, 12] and to induce the differentiation of Treg cells and inhibit Th17 cell function [13, 14] to exert their immunomodulatory effects in RA. Moreover, evidence in recent years has implied that bone marrow MSCs in RA may be involved in the disease pathogenesis [15]. However, it still needs to be elucidated whether intrinsic bone marrow MSCs are functionally altered in patients with RA. In this study, the function of MSCs from RA patients (RA MSCs) was characterized, focusing on both biological properties and immunomodulatory potential.

## 2. Materials and Methods

### 2.1. Bone Marrow MSC Culture

Eight RA patients (all female, aged 47~68 years) undergoing total knee arthroplasty were enrolled in this study, and six patients with osteoarthritis (all female, aged 55~76 years) undergoing total knee arthroplasty were recruited as controls. All RA patients fulfilled the 1987 revised diagnostic criteria of the American College of Rheumatology for RA [16] (Table 1). Bone marrow cells collected from discarded material of trabecular bone chips were treated with Red Blood Cell Lysis Solution (Miltenyi Biotec) and seeded at 10^5^/mL density in Dulbecco Modified Eagle Medium (DMEM)/F-12 (Gibco) supplemented with 10% Fetal Bovine Serum (FBS; Gibco) and 1% Penicillin-Streptomycin (Gibco). Cells were incubated at 37°C in a 5% humidified CO_2_ chamber, recovered by 0.25% trypsin-ethylenediaminetetraacetic acid (EDTA) (Gibco), and replanted when grown up to 80% confluency. Cells at passage 3–5 were used in our experiments. MSC phenotype was identified using the following antibodies (Abs) (eBioscience): CD14, CD34, CD45, CD31, CD44, CD73, CD105, and CD166. For differentiation, MSCs were cultured in osteogenic or adipogenic differentiation medium (Lonza). After 3 weeks, cells were stained with Alizarin Red or Oil Red O (Sigma-Aldrich), respectively. Expressions of the osteogenic marker runt-related transcription factor 2 (*Runx2*) and adipogenic marker peroxisome proliferator-activated receptor gamma (*PPARγ*) were measured by real-time polymerase chain reaction (PCR).

### 2.2. PBMC and MSC Coculture

MSCs were allowed to adhere to 24-well plates overnight. PBMCs were isolated from healthy donors by Ficoll-Hypaque density gradient (1.077 g/mL) (lymphoprep) and labeled with 5 *μ*M carboxyfluorescein diacetate succinimidyl ester (CFSE; eBioscience). Labeled PBMCs were resuspended in RPMI 1640 medium (Gibco) with 10% FBS (complete 1640 medium) and cocultured with MSCs at 10 : 1 with the stimulation of 5 *μ*g/mL anti-CD3/CD28 antibodies (eBioscience). After 4 days, PBMCs were harvested for the detection of CFSE fluorescence by flow cytometry. Proliferation index was calculated by Modfit LT Version 3.2 software (Verity Software House). In PBMC and MSC coculture system (10 : 1), floating cells were also collected after 4 days to examine the percentage of Tfh cells (CD4^+^CXCR5^+^PD-1^+^) by flow cytometry.

### 2.3. CD4^+^ T Cell and MSC Coculture

CD4^+^ T cells were isolated from healthy donors' PBMCs by human CD4 microbeads (Miltenyi Biotec) and resuspended in complete 1640 medium in the presence of anti-CD3/CD28 antibodies (5 *μ*g/mL), anti-IL4 antibody (10 *μ*g/mL) (eBioscience), and anti-IFN*γ* antibody (10 *μ*g/mL) (eBioscience). MSCs were cocultured with CD4^+^ T cells (1 : 10) in different culturing systems. For Treg induction, recombinant human TGF-*β*1 (5 ng/mL) (R&D Systems) and IL-2 (5 ng/mL) (Miltenyi Biotec) were added. For Th17 induction, recombinant human TGF-*β*1 (5 ng/mL), IL-6 (50 ng/mL) (Miltenyi Biotec), and IL-23 (10 ng/mL) (Miltenyi Biotec) were added. In some experiments anti-CCL2 antibody (R&D Systems) was added for neutralization. After coculture for 5 days, floating cells were used to examine Treg and Th17 cell percentages by flow cytometry, and culture supernatant was collected for measuring IL-17A levels by enzyme-linked immunosorbent assay (ELISA) kits (Biolegend). The adherent MSCs were also collected for measuring the gene expression of transforming growth factor- (TGF-) *β*1, indoleamine 2,3-dioxygenase (IDO), prostaglandin E2 (PGE2), interleukin- (IL-) 6, and chemokine (C-C motif) ligand 2 (CCL2) by real-time PCR.

### 2.4. Flow Cytometry

The following antibodies were used for surface staining: anti-human CD4-FITC, CD25-APC, PD1-Percp, and CXCR5-APC (eBioscience). For intracellular IL-17A staining, we stimulated cells for 5 hours with phorbol 12-myristate 13-acetate (PMA) (50 ng/mL), ionomycin (1 *μ*g/mL), and brefeldin A (5 *μ*g/mL) (Enzo). Cells were then stained with anti-human IL17A-APC (eBioscience) using a Fixation/Permeabilization Kit (MUbio). For transcription factor FoxP3 expression, staining was performed using anti-human FoxP3-PE with FoxP3 staining buffer (eBioscience). Data were acquired using a FACS calibur system (BD Biosciences) and analyzed by FlowJo software.

### 2.5. Proliferation and Apoptosis Assays

For measuring cell growth, MSCs from the two groups (RA and control) were seeded into 96-well plates (2 × 10^3^ cells/well). Proliferation potential of the cells was quantified using a cell counting kit (WST-8). On days 1, 3, 5, and 7, 10 *μ*L WST-8 solution was added to 100 *μ*L supernatant and incubated for 2 hours at 37°C. The optical density (OD) of supernatants was measured at 450 nm. After culture for 24 hours, cell apoptotic status was determined by flow cytometry using 7-aminoactinomycin D (7AAD; BD Biosciences). Expression of p21 was assayed by western blot analysis.

### 2.6. Migration Assays

MSC migration assays were conducted in 24-well plates. The contents of the upper and lower chambers were separated by Millicell Cell Culture Inserts (Millipore). MSCs (5 × 10^4^ cells) were resuspended in DMEM/F-12 without FBS and seeded in the upper wells. DMEM/F-12 with 10% FBS was added to the lower wells. After 24 hours of incubation, cells that had migrated through the membrane were fixed with 2% paraformaldehyde and stained with crystal violet (Sigma-Aldrich) and counted in five high power fields per membrane under light microscopy (Olympus). The results were presented as the average number of cells migrated per field. The gene expression of focal adhesion kinase (FAK), integrin *β*1, hepatocyte growth factor (HGF), vascular endothelial growth factor (VEGF), and C-X-C chemokine receptor type 4 (CXCR4) was analyzed by real-time PCR.

### 2.7. Protein Array Analysis

To analyze constitutive cytokine secretion, MSCs were plated at confluent cell concentrations (1 × 10^5^ cells/well) in 24-well plates. After culture for 24 hours, the supernatant was collected for cytokine determination by Human G-Series Cytokine Antibody Array (RayBiotech), which detects 60 human cytokines in one experiment. The signals were detected using a laser scanner (Innopsys' InnoScan). The signal intensity data were analyzed with the RayBio Analysis Tool software. Levels of IL-6, CCL2, and the regulated upon activation normal T cell expressed and secreted (RANTES) were further verified by ELISA.

### 2.8. RNA Isolation and Real-Time PCR

Total RNA was extracted from cells using Trizol (TaKaRa) and reverse-transcribed by PrimeScript RT Master Mix (TaKaRa). Quantitative real-time PCR assays using gene-specific primers (Table 2) and SYBR Premix Ex Taq kit (TaKaRa) were run on the StepOnePlus Real Time PCR Systems (Applied Biosystems). The relative expressions of each gene were determined and normalized to the expression of housekeeping gene glyceraldehyde 3-phosphate dehydrogenase (*GAPDH*). Relative quantification was calculated using 2^−ΔΔCT^ method.

### 2.9. Statistical Analysis

Data were presented as mean ± SEM and analyzed by Student's *t*-test or one-way analysis of variance (ANOVA) using SPSS 16.0 software or GraphPad Prism 5. *P* values less than 0.05 were considered to be statistically significant.

## 3. Results

### 3.1. RA MSCs Produced Low Level of CCL2 and Consequently Failed to Downregulate Th17 Cells

By flow cytometry analysis, bone marrow derived MSCs were positive for CD44, CD73, CD166, and CD105 and negative for CD14, CD45, CD34, and CD31 (Figure S1 in Supplementary Material available online at http://dx.doi.org/10.1155/2015/284215). The ability of RA MSCs to differentiate into osteogenic or adipogenic lineages was indistinguishable from that of controls as shown by cytochemical staining and expression of* Runx2* and* PPARγ* (Figure S2). These results confirmed the stem cell properties of RA MSCs.

**Figure 1:**
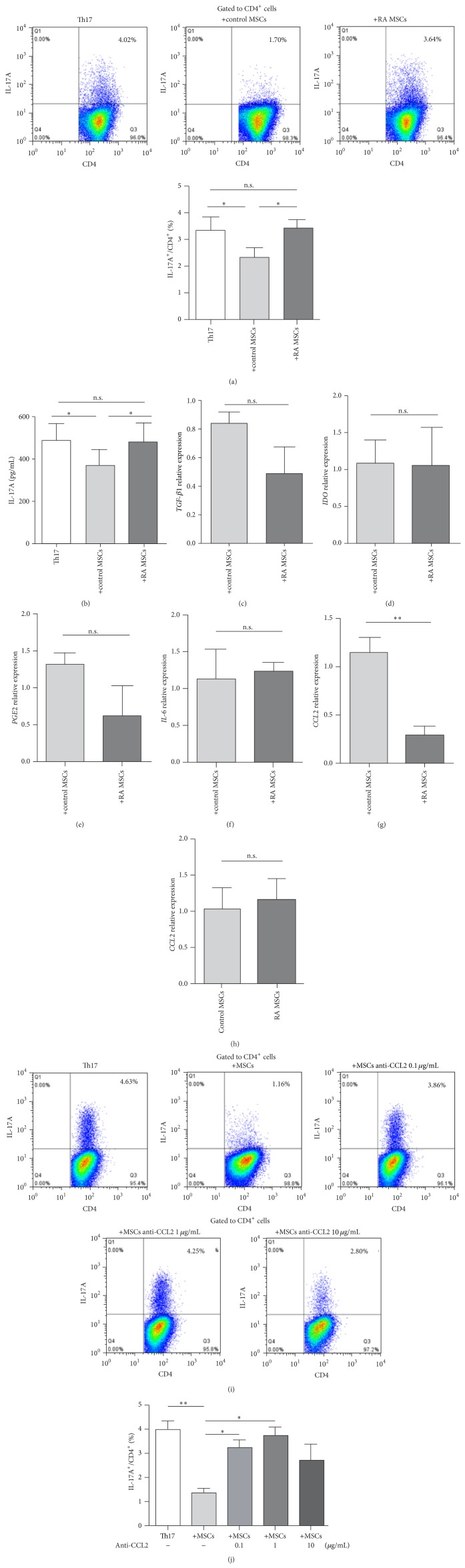
Impaired capacity of RA MSCs to inhibit Th17 cell polarization. (a) Flow cytometric analysis of IL-17A^+^CD4^+^ T cells under Th17-polarizing conditions with or without MSCs (*n* = 4 in each group). Percentages of Th17 subset are shown as mean ± SEM. (b) Production of IL-17A in cultured supernatants at day 5. (c–g) mRNA levels of immunomodulatory molecules TGF-*β*1, IDO, PGE2, IL-6, and CCL2 between control MSCs and RA MSCs after coculture with T cells. (h) Basal expressions of CCL2 in control MSCs and RA MSCs. (i-j) Percentages of IL-17A^+^CD4^+^ T cells after blocking CCL2 with neutralizing antibody at different concentrations in control MSC and CD4^+^ T cell coculture system under Th17-polarizing condition. ^∗^ *P* < 0.05; ^∗∗^ *P* < 0.01; n.s., no significant difference.* TGF-β1*, transforming growth factor *β*1;* IDO*, indoleamine 2,3-dioxygenase;* PGE2*, prostaglandin E2;* CCL2*, chemokine (C-C motif) ligand 2.

Because Th17 cells play a central role in RA pathogenesis, we compared the ability of control MSCs and RA MSCs on Th17 polarization using MSC and CD4^+^ T cell coculture system. From the results, the capacity of RA MSCs to inhibit Th17 cell induction was significantly impaired compared to that of control MSCs (Figure 1(a)). The protein level of IL-17A was also higher in RA MSC and CD4^+^ T cell coculture supernatant (Figure 1(b)). To clarify the molecular mechanism by which RA MSCs displayed the compromised effect on suppressing Th17 cells, we assessed the mRNA levels of several factors that have been reported to be involved in MSC-mediated Th17 cell regulation, including TGF-*β*1, IDO, PGE2, IL-6, and CCL2 [14, 17–19]. We found that there were no significant differences of TGF-*β*1, IDO, PGE2, and IL-6 expressions between control MSCs and RA MSCs after coculture with T cells (Figures 1(c)–1(f)). However, the mRNA levels of CCL2 were significantly lower in RA MSCs compared with control MSCs after coculture with T cells (*P* < 0.01) (Figure 1(g)). Our data also showed that the basic expression of CCL2 was comparable between RA MSCs and control MSCs (Figure 1(h)), indicating the differentially expressed CCL2 in RA MSCs after coculture may result from the interaction with T cells.

To further verify that CCL2 contributed to MSC-mediated Th17 inhibition, an anti-CCL2 neutralization antibody was added in MSC and CD4^+^ T cell coculture system at various concentrations. We found that anti-CCL2 treatment impaired the ability of MSCs to downregulate Th17 cells (Figures 1(i)-1(j)). Therefore, RA MSCs display defects in the inhibition of Th17 cell polarization, which is related to low expression of CCL2 when cocultured with T cells.

### 3.2. The Capacity of RA MSCs to Modulate Tfh and Treg Cells Remained Unchanged

**Figure 2:**
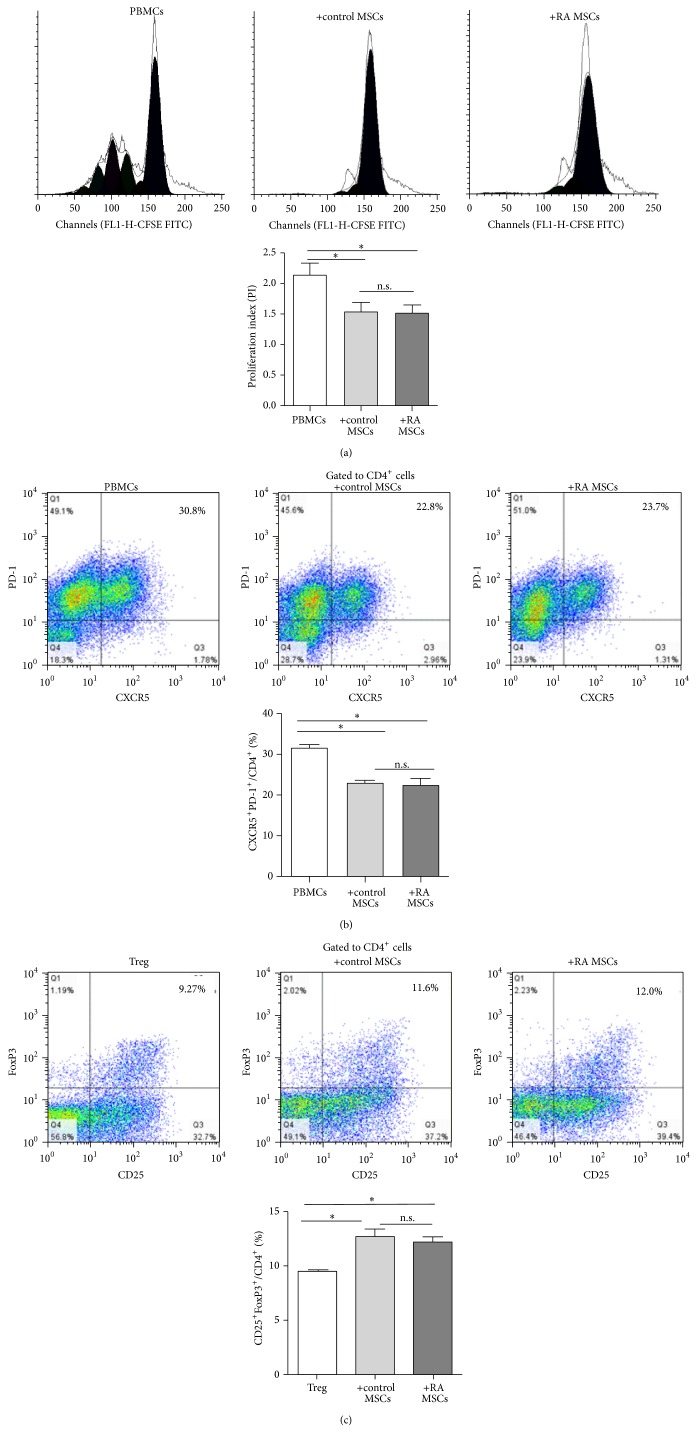
Immunomodulatory function of RA MSCs on Tfh and Treg cells. (a) The effect of MSCs on PBMC proliferation. CFSE-labelled PBMCs (5 × 10^5^ cells/well) were cultured 4 days under the stimulation of anti-CD3 and anti-CD28 antibodies with or without the presence of MSCs (5 × 10^4^ cells/well). For each sample (*n* = 4 in each group), a proliferation index is calculated. (b) Percentages of CXCR5^+^PD-1^+^ cells in CD4^+^ T cells (Tfh cells) were significantly downregulated by control MSCs or RA MSCs (*n* = 3 in each group). (c) Polarization of Treg cells from CD4^+^ T cells was significantly upregulated by control MSCs or RA MSCs (*n* = 3 in each group). ^∗^ *P* < 0.05; n.s., no significant difference. PBMCs, peripheral blood mononuclear cells; CFSE, carboxyfluorescein diacetate succinimidyl ester.

To evaluate the effect of MSCs on PBMC proliferation, the CFSE experiment was conducted. MSCs and lymphocytes were cocultured at the ratio of 1 : 10, which has been demonstrated to be effective for MSC-mediated immunosuppression [11]. At this ratio, MSCs from both RA patients and controls were able to suppress the proliferation of PBMCs at a comparable level (Figure 2(a)). Because CD4^+^ T helper cells are the key participant in RA pathological process and MSCs prominently regulate CD4^+^ T helper cells to exert their therapeutic effect in RA treatment, the function of RA MSCs on CD4^+^ T cell subtypes was further evaluated. We measured the Tfh cell (CD4^+^CXCR5^+^PD-1^+^) proportion in PBMC and MSC coculture system and detected the effects of MSCs on Treg cell (CD4^+^CD25^+^FoxP3^+^) polarization in CD4^+^ T cell and MSC coculture system. Our data showed that RA MSCs efficiently downregulated the proportion of Tfh cells (Figure 2(b)) and promoted the polarization of Treg cells (Figure 2(c)) as for control MSCs. Thus, MSCs may not act through the modulation of Tfh or Treg cells to participate in the pathogenesis of RA.

### 3.3. RA MSCs Showed Impaired Proliferative Potential and Migration Capacity

**Figure 3:**
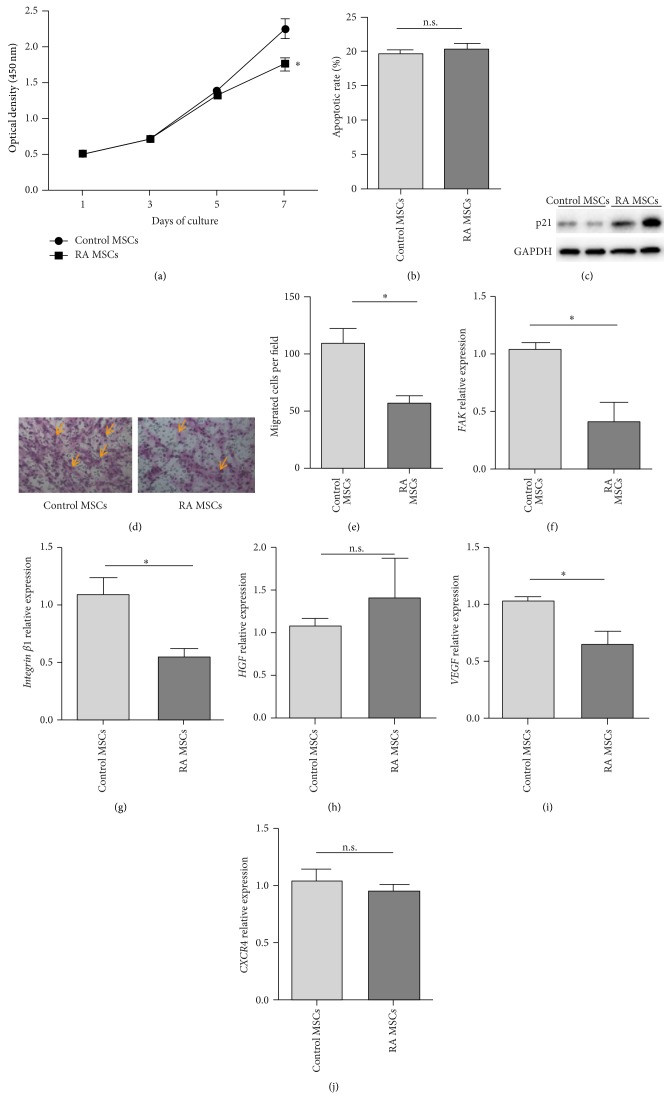
Proliferative potential, cellular apoptosis, and migration ability of RA MSCs. (a) Proliferation curves of control MSCs and RA MSCs. Optical density was measured at 450 nm. The graph represents the mean ± SEM of 3 controls versus 3 RA patients. (b) Apoptotic rate of control MSCs and RA MSCs (*n* = 3 in both groups). (c) Protein expression of p21 in control MSCs and RA MSCs. (d) Representative images of MSC migration as determined by transwell assay (crystal violet staining, magnification ×200). Some of the migrated cells are illustrated by yellow arrows. (e) Quantification of migrated cells per field (*n* = 3 in both groups). (f–j) mRNA levels of focal adhesion kinase (FAK), integrin *β*1, hepatocyte growth factor (HGF), vascular endothelial growth factor (VEGF), and C-X-C chemokine receptor type 4 (CXCR4) in control MSCs and RA MSCs (*n* = 4 in both groups). ^∗^ *P* < 0.05; n.s., no significant difference.

We next investigated the proliferation, apoptosis, and migration status of RA MSCs. The proliferative potential of MSCs was evaluated in 7 days of culture by the WST-8 assay. At day 7, the OD value was lower in RA MSCs than that in control MSCs (1.76 ± 0.09 versus 2.25 ± 0.13, *P* < 0.05) (Figure 3(a)). However, no significant differences in the percentages of apoptotic cells were observed between control MSCs and RA MSCs (Figure 3(b)). After culture for 24 hours, the number of migrated cells in RA MSCs was significantly lower than that in controls (56.8 ± 13.3 per field versus 109.3 ± 26.1 per field, *P* < 0.05) (Figures 3(d)-3(e)). These results suggested that RA MSCs appear to have deficient proliferation and migration capacity but normal apoptotic rate when compared to control MSCs.

p21 is a potent cyclin-dependent kinase inhibitor and functions as a regulator of cell cycle progression [20]. Western blot analysis showed higher expression of p21 in RA MSCs than control MSCs (Figure 3(c)). With regard to cell migration, focal adhesion kinase (FAK) is a nonreceptor tyrosine kinase that plays a key role at focal adhesion sites by promoting cell migration. Integrin *β*1 is known to activate FAK at the adhesive stage [21]. The mRNA levels of both FAK and Integrin *β*1 were decreased in RA MSCs compared to control MSCs (Figures 3(f)-3(g)). We also detected the expression of hepatocyte growth factor (HGF), vascular endothelial growth factor (VEGF), and C-X-C chemokine receptor type 4 (CXCR4) which have been reported to be associated with MSC migration [22]. Only VEGF mRNA level was decreased in RA MSCs compared to control MSCs (Figures 3(h)–3(k)). Collectively, the altered expression of FAK, integrin *β*1, and VEGF may be involved in the defective migratory capacity of RA MSCs.

### 3.4. RA MSCs Showed Comparable Cytokine Profiles to Control MSCs

**Figure 4:**
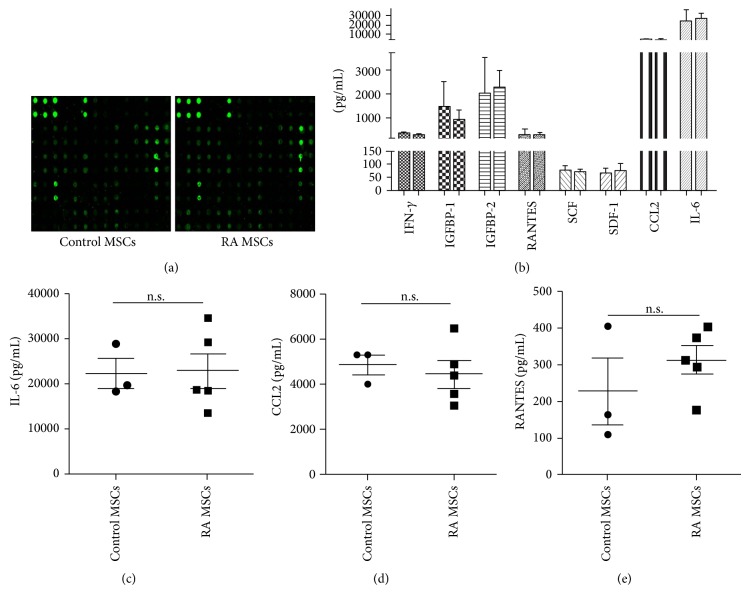
Protein array analysis of cytokine profiles in RA MSCs. After culture for 24 hours, MSC supernatant was collected and analyzed using a cytokine protein array. (a) The images of spot signal on the membrane due to each cytokine are shown. Detailed information of the cytokine protein array used in this study was shown in the supplementary material (Figure S3). (b) Histogram of several cytokines measured by the protein array. For data set of each cytokine, left column represents control MSCs and right column represents RA MSCs. (c–e) Levels of IL-6, CCL2, and RANTES assayed by ELISA. n.s., no significant difference. IGFBP, insulin-like growth factor binding protein; SCF, stem cell factor; SDF-1, stromal cell-derived factor-1; RANTES, the regulated upon activation normal T cell expressed and secreted, also called CCL5.

To determine the cytokine expression pattern of unstimulated RA MSCs, we collected the supernatants of cultured MSCs from five RA patients and three control patients and measured the levels of cytokines and chemokines using a protein array (Figure S3). All the factors measured, including interferon- (IFN-) *γ*, IL-1*α*, IL-1*β*, IL-2, IL-6, IL-7, IL-10, leptin, stem cell factor (SCF), stromal cell-derived factor-1 (SDF-1), insulin-like growth factor-binding protein (IGFBP), macrophage colony-stimulating factor (M-CSF), CCL2, RANTES (also called CCL5), TGF-*β*1, TGF-*β*3, and tumor necrosis factor- (TNF-) *α*, showed similar levels between the two groups (Figures 4(a)-4(b)). We also measured the secreting level of IL-6, CCL2, and RANTES by ELISA (Figures 4(c)–4(e)), and the data were consistent to those in protein array. These results suggested that the cytokine profiles in RA MSCs remain largely unchanged compared to control MSCs.

## 4. Discussion

Besides the fact that there are clearly defined disturbances of the immune system in RA, evidence in recent years has suggested that bone marrow may also be involved in the pathogenesis of RA. Abnormalities of both haemopoietic progenitor cells (HSCs) and MSCs in bone marrow of RA have been described in previous studies [15]. It was reported that patients with active RA exhibit a defect in MSC-mediated support of haematopoiesis [23]. In RA-like disease, a decrease in the number of mesenchymal progenitors in the bone marrow niche was also found to occur with the establishment and progression of disease [24]. Moreover, contrasting results have been reported for applying MSCs in treating RA. Some studies showed that MSCs are able to decrease arthritis in RA animal models; but in other studies, the immunosuppressive effect of MSCs might be turned off or even switched to stimulatory effect [25, 26]. Therefore, the role of MSCs in RA remains unclear in many aspects, and it calls for a closer analysis of the intrinsic function of MSCs in the setting of RA. To clarify this, we studied biological properties and especially the immunomodulatory potential of RA MSCs.

Since CD4^+^ T cell subtypes including Tfh, Treg, and Th17 cells have key roles in the pathogenesis of RA, we mainly compared the immunosuppressive properties of RA MSCs to their controls on the distribution of CD4^+^ T cell subsets. Tfh cells are a subtype of CD4^+^ T helper cells that regulate the development of B-cell immunity. Previous study showed that MSC treatment could significantly decrease the proportion of Tfh cells* in vivo* [27]. In this study, control MSCs significantly decreased the percentage of Tfh cells in MSC and PBMC coculture system, but the inhibitory effect of RA MSCs on Tfh cells was equally effective as controls. Similarly, the immunomodulatory function of RA MSCs to promote Treg cells also showed no differences as compared to controls. Besides that, our results also demonstrated the MSCs from RA patients have an antiproliferative effect on stimulated PBMCs as control MSCs, which was consistent with the previous studies in RA [28]. Previous studies showed that MSCs from systemic sclerosis patients could also effectively reduce PBMC proliferation as MSCs from healthy donors [29], but lupus patients derived MSCs failed to exert this effect [30]. Lupus patients derived MSCs showed defect in suppressing lymphocyte proliferation and this is in part attributed to their reduced ability to produce indoleamine 2,3-dioxygenase (IDO) in response to IFN-*γ*. However, the ability of RA MSCs to produce IDO appeared comparable to control MSCs (unpublished data). MSCs from different autoimmune diseases have distinct immunomodulatory function and this may be caused by the different pattern of inflammatory cytokines and immune cells.

Th17 cells may induce the production of chemokines and proinflammatory cytokines from stromal cells and stimulate matrix metalloproteinases from macrophages and stromal cells to maintain the self-perpetuating chronic inflammation; thus they play an important role in RA pathogenesis [31]. Our results showed that RA MSCs retained the capacity to regulate Tfh and Treg cells yet had a deficiency in the downregulation of Th17 cell polarization, supporting a fundamental effect of Th17 cells in RA patients. It has been reported that MSCs can inhibit Th17 cells via the secretion of CCL2, TGF-*β*1, or PGE2 [17, 19, 32, 33]. However, in RA MSCs, only the level of CCL2 was significantly declined after coculture with T cells. Previous studies showed MSCs inhibited experimental autoimmune encephalomyelitis- (EAE-) derived CD4^+^ T cell activation by suppressing STAT3 phosphorylation via CCL2, and CCL2^−/−^ MSCs could not ameliorate disease and decrease Th17 cells in EAE mice [19]. From our results, after anti-CCL2 antibody treatment, control MSCs also lost their capacity to modulate Th17 cells* in vitro* (Figure 1), suggesting lack of CCL2 production may weaken the regulation capacity of RA MSCs on Th17 cells. Collectively, the diverse effects of RA MSCs on different CD4^+^ T cell subsets suggest heterogeneity of MSCs in terms of immune functions, and the interactions between MSCs and immune cells still need to be further investigated. Recent studies have already shown that the immunosuppressive capacity of MSCs is not always achieved [34], and the function of MSCs probably depends on the specific inflammatory milieu of arthritis [25].

From our results RA MSCs showed similar immunophenotype, differentiation potential, cellular apoptosis, and cytokine profiles compared to controls; however, they displayed impaired capacity of proliferation and migration. It remains unclear why RA MSCs have impaired capacity of proliferation and migration. In our results, RA MSCs showed enhanced expression of p21, which is one of cell cycle inhibitors. p21 can mediate cell growth arrest at G1 and S phase of cell cycle progression [35]. In addition to that, RA MSCs have also been reported to have decreased cellular telomere, the length of which is closely associated with cell replicative capacity [36]. The altered expression of cell cycle associated protein p21 and inappropriate telomere loss may contribute to the defective proliferative potential in RA MSCs. In the aspect of cell migration, the dynamic assembly and disassembly of focal adhesions play a key role [37]. Previous study showed RA MSCs display altered expression of genes in focal adhesion [36]. In our study we confirmed the decreased expression of focal adhesion kinase (FAK), integrin *β*1, and vascular endothelial growth factor (VEGF) in RA MSCs. Integrins are known to act as linker between FAK and the actin cytoskeleton to promote cell migration [21]. VEGF has also been proved to be important for promoting MSC mobilization [38]. The reduced expression of these molecules in RA MSCs may account for the decreased migration capacity in these cells. Moreover, the impairment of both cell proliferation and migration may further cripple the immunomodulatory potential of RA MSCs, particularly in the local inflammatory sites.

When interpreting the results of this study, one should be alerted that the control MSCs applied here are derived from osteoarthritis patients because it is very hard to get the bone marrow cells from a truly healthy person. The function of osteoarthritis MSCs may be different from that of healthy donors, as inflammation of joints can also occur in osteoarthritis patients, though often mild compared to what occurs in RA. Theoretically, RA MSCs may be more “pathogenic” when compared to normal MSCs.

In conclusion, our results show that MSCs from RA patients have some abnormalities compared to those in controls, especially the capacity to inhibit Th17 cell polarization. Those abnormalities may be the consequence or the constitution of the RA pathological environment. Furthermore, the studies on the role of MSCs in RA pathological conditions are believed to be meaningful for a better understanding of the pathogenesis of RA and also helpful for harnessing the therapeutic potential of allogeneic MSC transplantation in RA.

## Supplementary Material

Bone marrow derived MSCs were positive for CD44, CD73, CD166, and CD105 and negative for CD14, CD45, CD34, and CD31 as shown by flow cytometry analysis (Figure S1). The ability of RA MSCs to differentiate into osteogenic or adipogenic lineages was indistinguishable from that of controls as shown by cytochemical staining and expression of Runx2 and PPAR?? (Figure S2). Cytokines for each dot in the protein array (in Result 3.4.) are shown in Figure S3.

## Figures and Tables

**Table 1:** Clinical and laboratory data of RA patients.

Patient	Age/sex	Duration (months)	ESR (mm/h)	CRP (mg/L)	DAS28
1	59/F	48	43	8.9	3.85
2	54/F	60	54	8.3	3.61
3	47/F	30	38	49.7	3.13
4	47/F	360	16	5.0	3.12
5	68/F	480	65	7.3	4.07
6	64/F	240	50	6.8	4.35
7	59/F	50	35	6.1	4.24
8	67/F	36	53	47.7	4.81

ESR, erythrocyte sedimentation rate; CRP, C reactive protein; DAS28, disease activity score in 28 joints.

**Table 2:** Gene-specific primers used for real-time PCR.

Gene	Sense primer	Antisense primer
*Runx2 *	5′-AGAGGTACCAGATGGGACTGT-3′	5′-GGTAGCTACTTGGGGAGGATT-3′
*PPARγ*	5′-TCGACCACGTCAATCCAGAGT-3′	5′-TCGCCTTTGCTTTGGTCAG-3′
*TGF-β1 *	5′-AGCGACTCGCCAGAGTGGTTA-3′	5′-GCAGTGTGTTATCCCTGCTGTCA-3′
*IDO *	5′-GAATGGCACACGCTATGGAA-3′	5′-CAGACTCTATGAGATCAGGCAGATG-3′
*PGE2 *	5′-TGACCAGAGCAGGCAGATGAA-3′	5′-CCACAGCATCGATGTCACCATAG-3′
*IL-6 *	5′-TGAAAGCAGCAAAGAGGCA-3′	5′-TGGGTCAGGGGTGGTTAT-3′
*CCL2 *	5′-GCTCATAGCAGCCACCTTCATTC-3′	5′-GGACACTTGCTGCTGGTGATTC-3′
*GAPDH *	5′-GCACCGTCAAGGCTGAGAAC-3′	5′-TGGTGAAGACGCCAGTGGA-3′
*FAK *	5′-GCCTTAACAATGCGTCAGTTTGACC-3′	5′-TCAGTGTGGTCTCGTCTGCCCAAG-3′
*Integrin β1 *	5′-GGGAAACTTGGTGGCATTG-3′	5′-GCTCCTTGTAAACAGGCTGAAA-3′
*HGF *	5′-GAAGGTGAAGGTCGGAGTC-3′	5′-GAAGATGGTGATGGGATTTC-3′
*VEGF *	5′-CCCTGATGAGATCGAGTACA-3′	5′-AGGAAGCTCATCTCTCCTAT-3′
*CXCR4 *	5′-CCTCCTGCTGACTATTCCCGA-3′	5′-GGAACACAACCACCCACAAGT-3′

*Runx2*, runt-related transcription factor 2; *PPARγ*, peroxisome proliferator-activated receptor gamma; *TGF-β1*, transforming growth factor-*β*1; *IDO*, indoleamine 2,3-dioxygenase; *PGE2*, prostaglandin E2; *IL-6*, interleukin-6; *CCL2*, chemokine (C-C motif) ligand 2; *GAPDH*, glyceraldehyde 3-phosphate dehydrogenase; *FAK*, focal adhesion kinase; *HGF*, hepatocyte growth factor; *VEGF*, vascular endothelial growth factor; *CXCR4*, C-X-C chemokine receptor type 4.
