# Supplementary material for: Mesenchymal Stem Cells from Patients with Rheumatoid Arthritis Display Impaired Function in Inhibiting Th17 Cells
Source: J Immunol Res. 2015 Mar 31;2015:284215. doi: 10.1155/2015/284215 (PMC4397051; doi:10.1155/2015/284215)
Supplement: Supplementary file 1 — Bone marrow derived MSCs were positive for CD44, CD73, CD166, and CD105 and negative for CD14, CD45, CD34, and CD31 as shown by flow cytometry analysis (Figure S1). The ability of RA MSCs to differentiate into osteogenic or adipogenic lineages was indistinguishable from that of controls as shown by cytochemical staining and expression of Runx2 and PPAR?? (Figure S2). Cytokines for each dot in the protein array (in Result 3.4.) are shown in Figure S3. [file 284215.f1.pdf]

Supplementary Materials

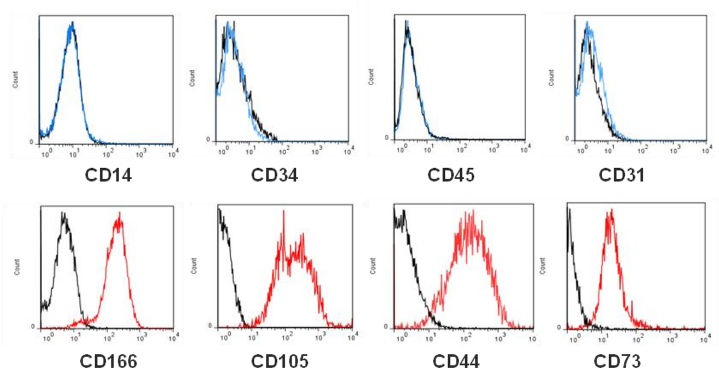

FIGURE S1: Immunophenotype of RA MSCs. Culture-expanded MSCs are comprised of a homogenous cell population positive for CD166, CD105, CD44, CD73 and negative for CD14, CD34, CD45, CD31 surface markers. The blue and red histograms depict the negative and positive markers respectively. Isotype control staining is represented by the black histograms.

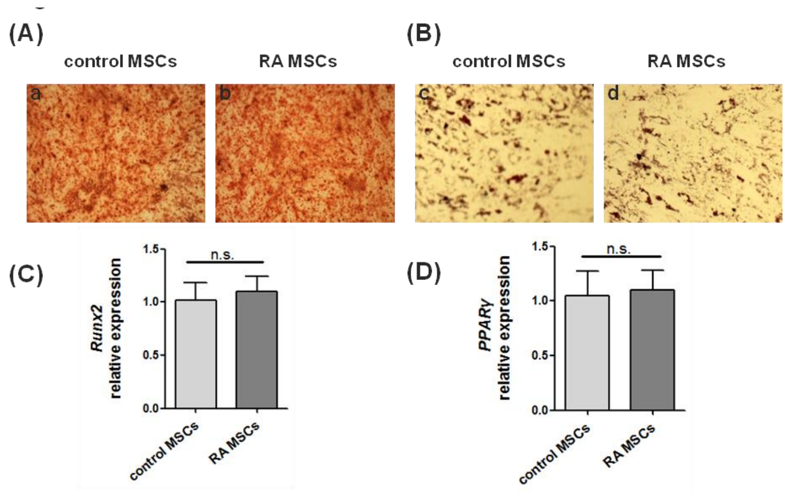

FIGURE S2: Differentiation potential of RA MSCs. (A) MSCs from controls (a) and RA patients (b) differentiated into osteoblasts (Alizarin red staining, magnification ×200). (B) MSCs from controls (c) and RA patients (d) differentiated into adipocytes (Oil Red O staining, magnification ×200). The figure shows one representative results from three independent experiments. (C-D) mRNA expression levels of the osteogenic marker *Runx2* and the adipogenic marker *PPARγ* on day 21 after osteogenic and adipogenic induction (n=3 for control MSCs and RA MSCs). n.s., no significant difference. *Runx2,* runt-related transcription factor 2; *PPARγ,* peroxisome proliferator-activated receptor gamma.

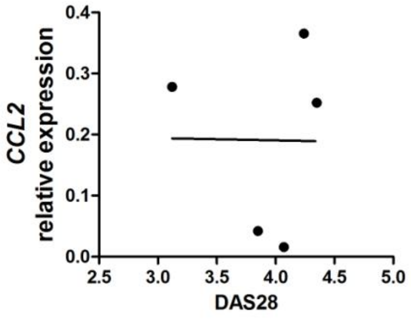

FIGURE S3: No significant correlation between DAS28 in RA patients with CCL2 expression in RA MSCs after co-culture. DAS28, disease activity score in 28 joints.

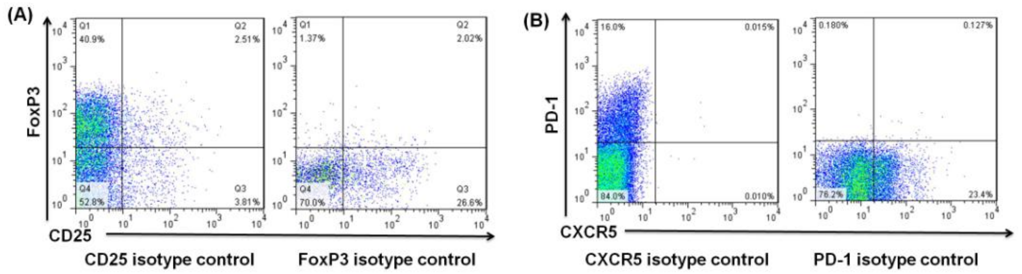

FIGURE S4: Isotype control staining of CD25, FoxP3, CXCR5 and PD-1.

	A	B	C	D	E	F	G	H	I	J	K	L	M	N
1	POS1	POS2	POS3	NEG	NEG	ANG	BDNF	BLC	BMP-4	BMP-6	Ckβ 8-1	CNTF	EGF	CCL11
2	POS1	POS2	POS3	NEG	NEG	ANG	BDNF	BLC	BMP-4	BMP-6	Ckβ 8-1	CNTF	EGF	CCL11
3	CCL24	CCL26	FGF6	FGF7	Flt-3L	CX3CL1	GCP-2	GDNF	CSF2	I-309	IFN-γ	IGFBP1	IGFBP2	IGFBP4
4	CCL24	CCL26	FGF6	FGF7	Flt-3L	CX3CL1	GCP-2	GDNF	CSF2	I-309	IFN-γ	IGFBP1	IGFBP2	IGFBP4
5	IGF-I	IL-10	IL-13	IL-15	IL-16	IL-1α	IL-1β	IL-1ra	IL-2	IL-3	IL-4	IL-5	IL-6	IL-7
6	IGF-I	IL-10	IL-13	IL-15	IL-16	IL-1α	IL-1β	IL-1ra	IL-2	IL-3	IL-4	IL-5	IL-6	IL-7
7	Leptin	LIGHT	MCP1	MCP2	MCP3	MCP4	M-CSF	MDC	MIG	MIP-1δ	MIP-3α	NAP-2	NT-3	PARC
8	Leptin	LIGHT	MCP1	MCP2	MCP3	MCP4	M-CSF	MDC	MIG	MIP-1δ	MIP-3α	NAP-2	NT-3	PARC
9	PDGF-BB	RANTES	SCF	SDF-1	TARC	TGF-β1	TGF-β3	TNF-α	TNF-β	NEG	NEG	NEG	NEG	NEG
10	PDGF-BB	RANTES	SCF	SDF-1	TARC	TGF-β1	TGF-β3	TNF-α	TNF-β	NEG	NEG	NEG	NEG	NEG

FIGURE S5: Cytokines for each dot in the protein array.

TABLE S1: C reactive protein (CRP) levels of osteoarthritis patients used in this study.

Patient	CRP (mg/L)
1	1.2
2	1.5
3	2.6
4	1.8
5	5.5
6	1.7
